# Field strength and frequency-dependent effects of pulsed electric fields on soilborne nematodes, pathogens, and weeds

**DOI:** 10.1038/s41598-026-56820-y

**Published:** 2026-06-08

**Authors:** Tatiana Benedetti, Pamela M. dos Santos, Inga A. Zasada, Jerry E. Weiland, Jason Crisp, Marcelo L. Moretti

**Affiliations:** 1 Department of Horticulture, Oregon State University, Corvallis, OR USA; 2 United States Department of Agriculture - Agricultural Research Service, Horticultural Crops Disease and Pest Management Research Unit, Corvallis, OR USA; 3 Lisi Global, Richland, WA USA

**Keywords:** *Cyperus esculentus*, *Digitaria sanguinalis*, *Echinochloa crus-galli*, *Meloidogyne chitwoodi*, *Phytophthora cinnamomi*, *Phytophthora plurivora*, *Pratylenchus neglectus*, *Verticillium dahlia*, Ecology, Ecology, Environmental sciences, Microbiology, Plant sciences

## Abstract

Pulsed electric field (PEF) technology is a non-chemical approach with potential to control soilborne agricultural pests. PEF disrupts cellular membranes through short, high-energy electrical pulses. Treatment efficacy depends on electric field strength, pulse frequency, and applicator design. Field strength reflects the intensity of the electric field driving electroporation, whereas energy density represents the cumulative dose delivered to the system, and pulse frequency determines how that dose is distributed over time. In this study, two soil-applied PEF applicators, vertical pins (VP) and parallel plates (PP), were evaluated for suppression of weeds, plant-parasitic nematodes, and soilborne pathogens. Numerical simulations showed that the PP configuration generated a more uniform electric field distribution than the VP design. Consistent with these predictions, the PP applicator provided greater biological suppression across target organisms. *Cyperus esculentus* biomass declined progressively with increasing energy density and was significantly reduced at ≥100 J cm^−3^. The PP applicator also reduced *Meloidogyne chitwoodi* second-stage juveniles by up to 100% at 25 J cm^−3^ and pulse frequencies above 60 Hz. Densities of *Pratylenchus neglectus* and *M. chitwoodi* were reduced by more than 98% at 400 V mm^−1^. Weed responses were species dependent: biomass of *C. esculentus* and *Digitaria sanguinalis* declined at 100–200 J cm^−3^, whereas *Echinochloa crus-galli* required higher energy inputs for control. Soilborne pathogens also differed in susceptibility, with *Phytophthora cinnamomi* and *P. plurivora* reduced by 94% at moderate field strengths (100–200 V mm^−1^), *while Verticillium dahliae* was suppressed by up to 97% at 200–400 V mm^−1^ and 80 Hz. Overall, nematodes and soilborne pathogens were suppressed at substantially lower energy densities (~25 J cm^−3^) than weed propagules, which generally required ≥100–200 J cm^−3^ for effective biomass suppression. These results demonstrate that PEF can effectively suppress multiple soilborne pests. Additionally, the results highlight the importance of applicator design and electrical parameters in determining treatment efficacy, supporting the potential of PEF as a scalable and sustainable soil disinfestation technology.

**Supplementary Information:**

The online version contains supplementary material available at 10.1038/s41598-026-56820-y.

## Introduction

Pulsed electric field (PEF) technology is an emerging strategy for agricultural pest management^1^. Many alternative chemical, biological, and physical approaches have been tested as replacements for traditional chemical fumigants^2^. PEF has been used in soil electrokinetic remediation, in the food processing industry, and in genetic engineering to inactivate bacteria, fungi, and yeasts^3,4^. More recently, it has demonstrated potential to control soilborne plant pathogens and plant-parasitic nematodes^5^.

Pulsed electric field delivers short, high-voltage electrical pulses of micro- to millisecond duration, creating intense electric fields across cell membranes. When the induced transmembrane potential exceeds a critical threshold, structural rearrangements occur within the lipid bilayer, leading to pore formation^6,7^. Electroporation can be reversible, allowing temporary membrane permeabilization, or irreversible, resulting in loss of cell viability. The outcome depends on field strength, pulse intensity, duration, number, and total energy input^8^. For example, PEF applied at an energy density of 25 J cm^−3^ to soil reduced *Meloidogyne hapla* egg viability, whereas an application of 70 J cm^−3^ reduced *Globodera ellingtonae* egg hatch by 85%^5^. At the same energy density, *Phytophthora cinnamomi* viability was reduced to nondetectable levels, while *Verticillium dahliae* viability was reduced by 73%^5^.

The electric field is established between at least two electrodes of opposite polarities separated by a defined physical distance. Electrode arrangement, geometry, spacing, surface area, and material strongly influence electric field uniformity, current-density distribution, energy delivery, and treatment uniformity^9,10^. Nonuniform field distribution within the treatment zone can leave portions of the treated volume below the electroporation threshold even when the cumulative delivered energy is high^11^. As PEF delivery systems continue to improve, further research is needed to assess new PEF configurations to manage soilborne pests.

In this study, field strength refers to the nominal voltage gradient between electrodes and is reported as V mm^−1^. Pulse duration refers to the on-time of each square-wave pulse. Pulse frequency refers to the number of pulses delivered per second and is reported in Hz. Energy density refers to the cumulative electrical energy delivered to the treated soil volume, calculated by integrating measured voltage and current over each pulse, summing across the pulse train, and normalizing by treated soil volume. Thus, field strength describes electrical intensity, frequency describes pulse timing and inter-pulse interval, and energy density describes cumulative delivered dose.

Plant-parasitic nematodes are among the most economically important soilborne pests in agricultural systems. Nematodes of importance to agriculture in the Pacific Northwest of the United States were considered^12^. *Meloidogyne chitwoodi*, a sedentary endoparasite, is a quarantine pest^13^. The damage that this nematode causes to tubers makes them unsaleable and rejection comes at a great cost to growers. In contrast, *Pratylenchus neglectus* is a migratory endoparasite of importance in the production of many different crops in the region, including potato and wheat. Similarly, *Phytophthora cinnamomi*, *Phytophthora plurivora*, and *Verticillium dahliae* represent major soilborne pathogens with diverse survival structures and infection strategies^14,15^*.* Weed species, including *Cyperus esculentus*, *Digitaria sanguinalis*, and *Echinochloa crus-galli*, are among the most problematic weeds in agriculture^16^.

Despite these advances, previous studies have largely evaluated PEF effects on individual organism groups or under limited combinations of electrical parameters. In particular, the combined influence of applicator design, field strength, pulse frequency, and energy density on biological responses in soil systems remains poorly understood. Furthermore, differences in susceptibility among weeds, plant-parasitic nematodes, and soilborne pathogens have not been systematically compared within a unified experimental framework. The present study evaluated two PEF applicator configurations, vertical pins (VP) and parallel plates (PP), to determine how electrode geometry influences electric field distribution and biological efficacy. We assessed the effects of field strength and pulse frequency on the suppression of weeds, plant-parasitic nematodes, and soilborne pathogens, and compared responses among these organism groups under controlled soil conditions. This approach provides a direct comparison of PEF performance across multiple soilborne pests under a common experimental framework.

## Results

The combined evaluation of energy density, field strength, pulse duration, and pulse frequency is important because these parameters influence biological responses differently. Field strength reflects electrical intensity, pulse frequency determines temporal exposure, and energy density represents the cumulative delivered dose normalized to treated soil volume. Evaluating these parameters together provides a more complete understanding of PEF effects across organism groups.

### Applicator comparison

**Fig. 1:**
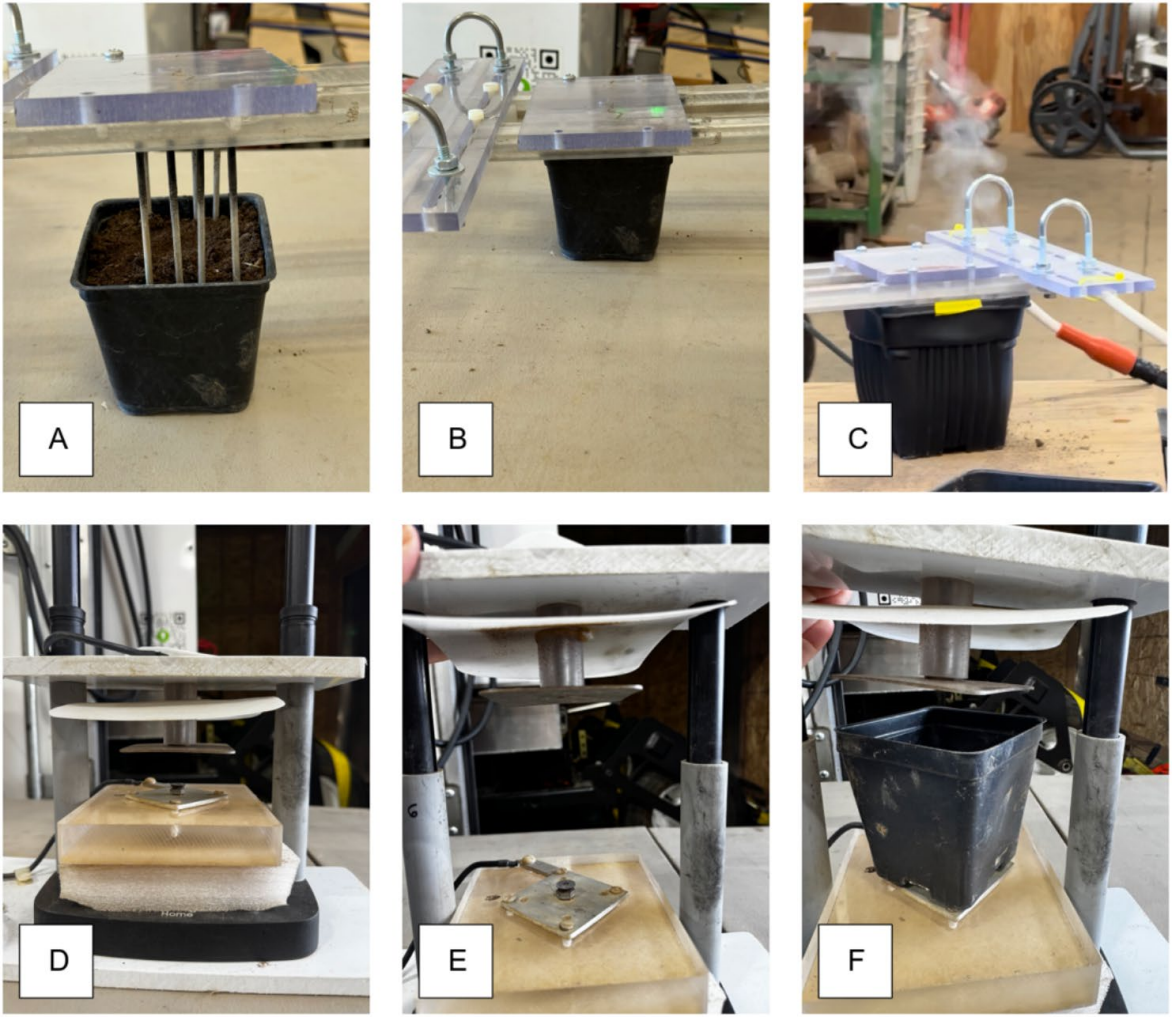
Pulsed electric field (PEF) application system used to treat weeds, plant-parasitic nematodes, and soilborne pathogens in soil-filled pots. The vertical pins applicator consisted of eight metal electrodes (0.32 cm diameter, 7.62 cm length) arranged in two rows of four, spaced 15 cm between rows and 1.27 cm within rows (**A**). The pins were fully inserted into the soil during treatment (**B**). Energy delivery was controlled by pulse number, field strength, and frequency (**C**). The parallel plate applicator was constructed using a modified mechanical frame to support electrode placement (**D**–**F**), with spring-loaded plates ensuring consistent soil–electrode contact during operation.

**Fig. 2:**
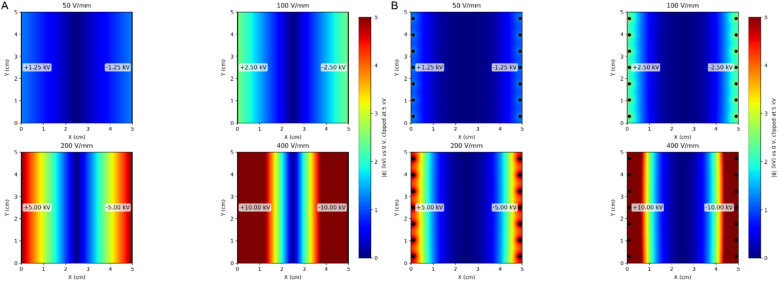
Simulated electric potential distribution for fixed representative parallel-plate and vertical-pin geometries. Simulations were conducted at field strengths of 50, 100, 200, and 400 V mm^−1^. Color scale represents electric potential magnitude |Φ| (kV), capped at ±10 kV. The parallel-plate configuration produces a more uniform nominal field across the inter-electrode region, whereas the vertical-pin configuration generates localized gradients near electrode surfaces.

Results from numerical simulations and biological assays showed that PEF applicator configuration (VP vs. PP) influenced electric-field distribution and, consequently, biological efficacy. Differences between the VP (Fig. 1A–C) and PP (Fig. 1D-F) configurations reflect their electrode geometry and resulting field distribution (Table 1). Numerical simulations of electric potential and nominal field magnitude showed clear geometry-driven differences between applicator configurations (Fig. 2). The VP configuration produced localized high-field regions near electrode surfaces that rapidly dissipated with distance (Fig. 2A), whereas the PP configuration generated a more uniform nominal field across the inter-electrode region (Fig. 2B). These differences are consistent with the larger electrode surface area to treated soil volume ratio of the PP applicator (A/V = 27.0 m^−1^) compared with the VP applicator (A/V = 14.1 m^−1^), resulting in a larger fraction of the treated volume being exposed to effective field intensities.

**Table 1:** Pulsed electric field treatment parameters applied for suppression of plant-parasitic nematodes, soilborne pathogens, and weed propagules.

*Species*	Treated pest stage or organ	Energy (J cm^−3^)	FS (V mm^−1^)	FQ (Hz)	PD (s)	App.	N. exp.
Applicator development							
*M. chitwoodi*	J2	25	100; 200	0–80	0.005	VP & PP	4
*P. neglectus*	mixed life stages	25	100; 200	0–80	0.005	VP & PP	4
*C. esculentus*	tuber	0–200	50; 250	100	0.005	VP & PP	4
*C. esculentus*	tuber	0–200	10; 800	100	0.001; 0.005	VP & PP	4
Weed species effect of field strength							
*E. crus-galli*	seed	0–200	50, 250	100	0.005	PP	2
*D. sanguinalis*	seed	0–200	50, 250	100	0.005	PP	2
Nematodes frequency and field strength							
*M. chitwoodi*	J2	25	200; 400	0–120	0.005	PP	4
*P. neglectus*	mixed life stages	25	200; 400	0–120	0.005	PP	2
Soil pathogens frequency and field strength							
*P. cinnamomi*	mixed life stages	25	100; 200	0–80	0.005	PP	2
*P. plurivora*	mixed life stages	25	100; 200	0–80	0.005	PP	2
*V. dahliae*	microsclerotia	25	100; 200	0–80	0.005	PP	2
*V. dahliae*	microsclerotia	25	200; 400	0–80	0.005	PP	2

VP = vertical pin; PP = parallel plate; N exp. = number of experiments conducted. FS – nominal field strength target; FQ – pulse frequency; PD – pulse duration; App – applicator type. Zero-frequency entries represent nontreated/no-pulse controls where applicable and should not be interpreted as active 0 Hz PEF treatment. For PP biological treatments, voltage was adjusted to achieve the prescribed nominal field strength for the corresponding treatment geometry, subject to the maximum DES output limit.

**Fig. 3:**
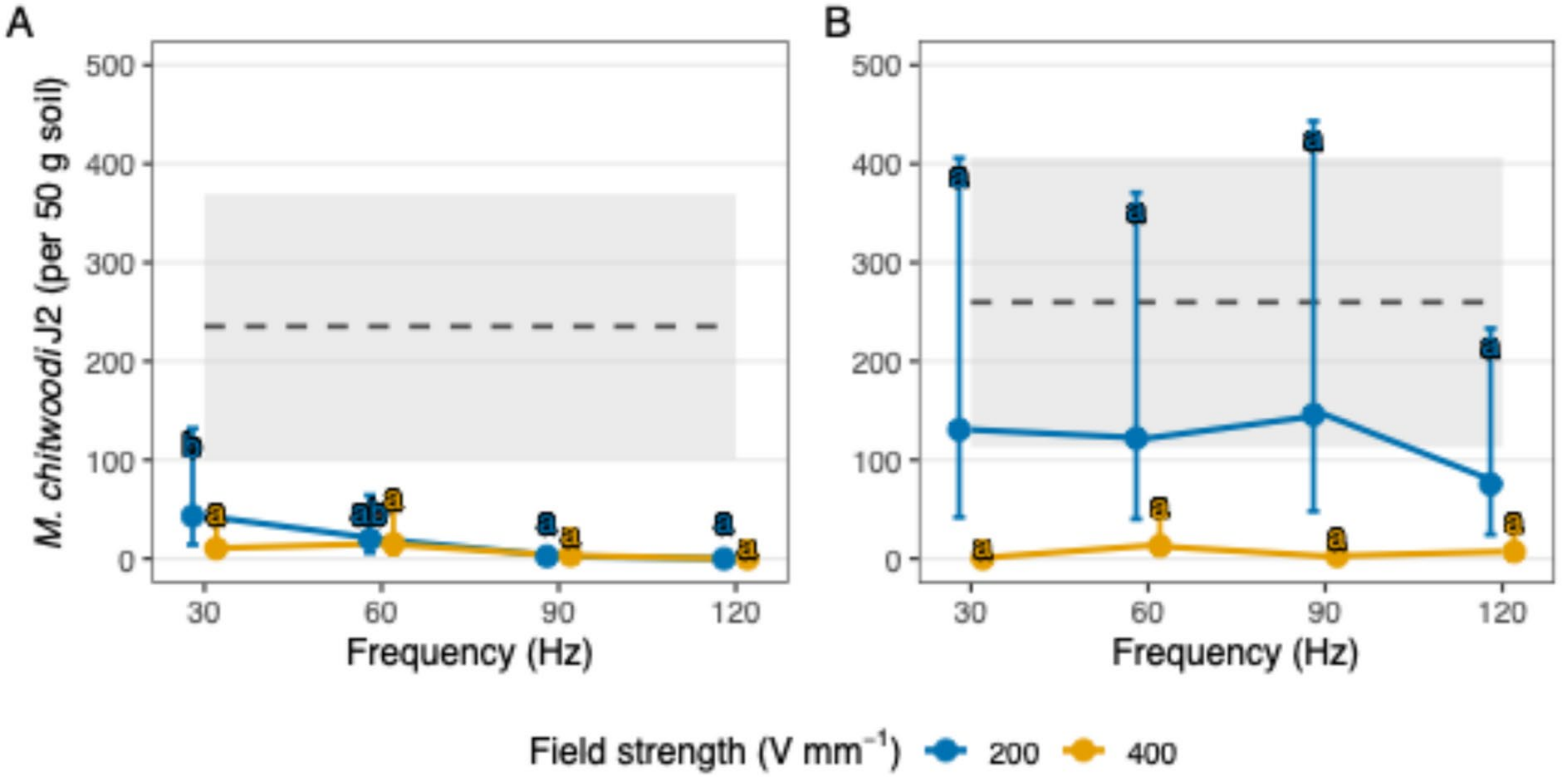
Response of *Meloidogyne chitwoodi* second-stage juveniles (J2) to PEF treatment at 25 J cm^−3^ applied at 200 and 400 V mm^−1^ using (**A**) parallel plate and (**B**) vertical pin applicators. Different letters indicate significant differences among treatments (*p* < 0.05). Model means ± SE (*n* = 8) are pooled from two studies. The dashed line and shaded area represent the mean and 95% confidence interval of the nontreated control.

**Fig. 4:**
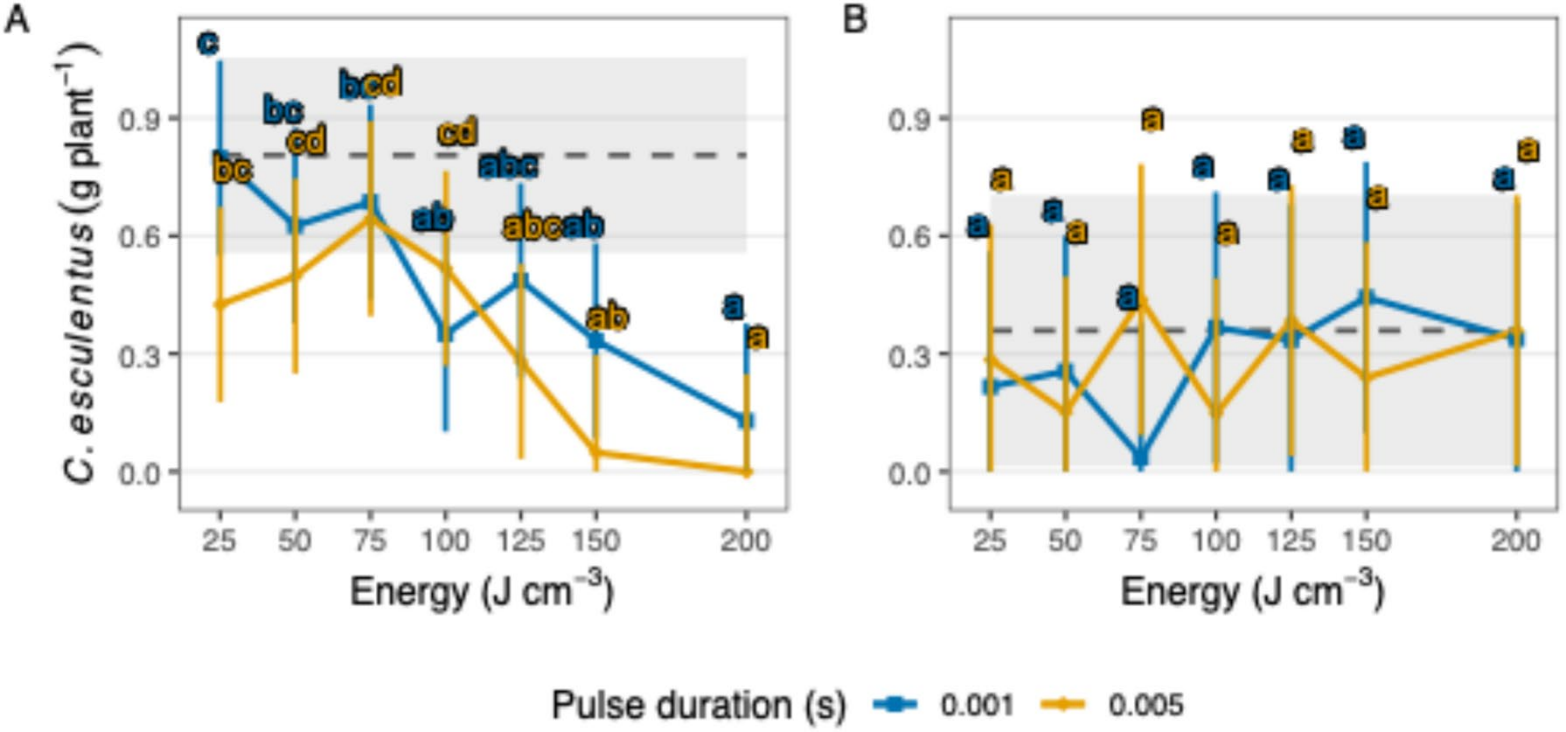
Response of *Cyperus esculentus* tubers to increasing energy densities (25–200 J cm^−3^) at pulse durations of 0.001 and 0.005 s using (**A**) parallel plate and (**B**) vertical pin applicators. Different letters indicate significant differences among treatments (*p* < 0.05). Means ± SE (*n* = 8) are pooled from two studies. The dashed line and shaded area represent the mean and 95% confidence interval of the nontreated control.

The applicators were subsequently compared for their effects on *M. chitwoodi* second-stage juveniles (J2) and *C. esculentus*. Differences between applicator types were observed for both nematode and weed control (Figs. 3 and 4; Table 2). Both applicators significantly reduced *M. chitwoodi* J2 densities relative to the nontreated control. The VP applicator consistently reduced nematode densities across all tested frequencies, whereas the PP applicator response was frequency-dependent, with significant reductions observed at ≥60 Hz (*p* < 0.05). At 60 Hz, reductions reached 91% with the PP applicator (Fig. 3A) and 73% with the VP applicator (Fig. 3B), indicating greater suppression with the PP configuration. Applicator performance also varied with field strength, with the PP applicator achieving complete suppression at higher frequencies, whereas the VP applicator required approximately twice the field strength to achieve comparable suppression. The greater efficacy of the PP applicator is consistent with its more uniform energy distribution and larger electrode–soil interface relative to the treated soil volume.

**Table 2:** Summary of analysis of variance for significant factors and interactions for pulsed electric field applications to *Meloidogyne chitwoodi* and *Cyperus esculentus* with different applicators.

*Meloidogyne chitwoodi*			*Cyperus esculentus*		
*Frequency (FQ)*			*Pulse Duration (PD)*		
*Factors*	χ^2^	*P*	*Factors*	χ^2^	*P*
*Field strength*	*67.03*	***	*Energy*	*66.24*	***
*Applicator*	*14.93*	***	*Applicator*	*19.47*	***
*FQ*	*12.94*	***	*PD*	*5.15*	*
Field strength *Applicator	*18.26*	**	Energy * Applicator	*45.85*	***
Applicator * FQ	*9.71*	**	Applicator * PD	*2.68*	*0.10*

P – Probability: 0 ‘***’ 0.001 ‘**’ 0.01 ‘*’ 0.05 ‘.’ 0.1 ‘ ’ 1

**Fig. 5:**
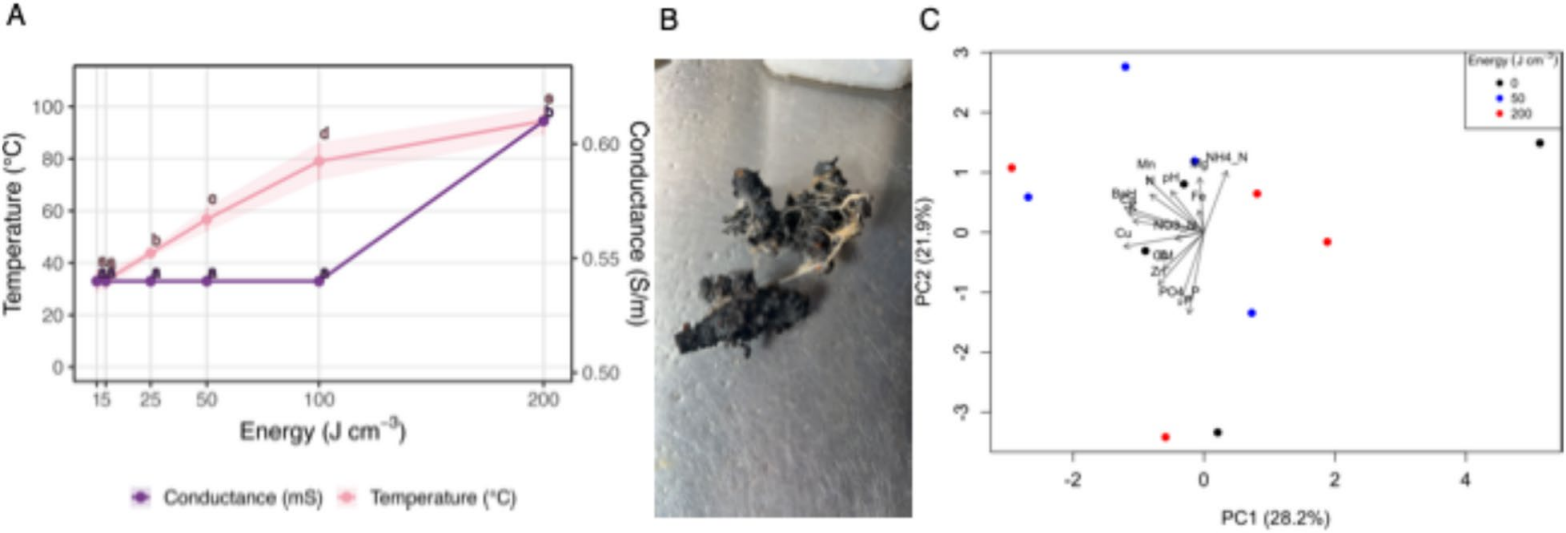
(**A**) Soil temperature (°C) and electrical conductivity (S m^−1^) following PEF application at 200 and 400 V mm^−1^ using the parallel plate applicator. Data represent means ± SE (*n* = 8). Different letters indicate significant differences among energy densities (*p* < 0.05). (**B**) Thermally induced soil induration was observed at the highest energy input (200 J cm^−3^). (**C**) Principal component analysis (PCA) of soil chemical attributes (Mehlich-3) following PEF application at 0, 50, and 200 J cm^−3^. No significant separation among treatments was observed (*p* > 0.05).

The effect of applicator type on *C. esculentus* was evaluated across energy densities ranging from 0 to 200 J cm^−3^ and two pulse durations (0.001 and 0.005 s) (Fig. 4 A,B). Using the PP applicator, plant biomass declined progressively with increasing energy density, with a stronger response observed at the greater pulse duration (0.005 s) (Fig. 4A). In contrast, the VP applicator produced more variable responses across energy densities, with greater variability among replicates (Fig. 4B). These differences are consistent with the heterogeneous electric-field distribution produced by the pin geometry, where field intensity and current density are concentrated near discrete electrode contact points rather than distributed uniformly throughout the treated soil volume. Overall, the PP applicator provided more consistent and effective suppression of plant growth compared with the VP configuration. At the energy levels used in this experiment, temperature increases were minimal (Fig. 5), indicating that suppression was primarily driven by electrical rather than thermal effects. Because of its higher and more consistent efficacy, subsequent experiments were conducted using the PP applicator (Table 1).

### Changes in soil physical and chemical properties to PEF treatment

Soil temperature increased with energy density, rising from approximately 35 °C at 15 J cm^−3^ to nearly 100 °C at 200 J cm^−3^ (Fig. 5A). At the highest energy densities (> 100 J cm^−3^), soil hardened, indicating thermally induced physical changes (Fig. 5B). In contrast, electrical conductivity remained stable up to 100 J cm^−3^ and increased sharply only at the higher energy densities. Statistical groupings confirmed that temperature differed significantly across energy densities, whereas conductivity differed only at the highest levels. These results indicate that temperature responds directly to increased energy input, whereas conductivity exhibits a threshold response at high-energy exposure.

The multivariate analysis of soil chemical attributes, based on Mehlich-3 extractions, indicated that PEF application (0, 50, and 200 J cm^−3^) did not significantly affect soil nutrient profile (Fig. 5C). Mehlich-3 extraction is a widely used method to quantify plant-available (extractable) nutrients in soil. No consistent separation among treatments was observed in PCA, and this result was supported by ANOVA of PCA scores and PERMANOVA (*p* = 0.944). Similarly, redundancy analysis (RDA) showed treatment centroids clustered near the origin, indicating no detectable effect of energy on soil nutrient availability.

### Effect of PEF on weed propagules

**Fig. 6:**
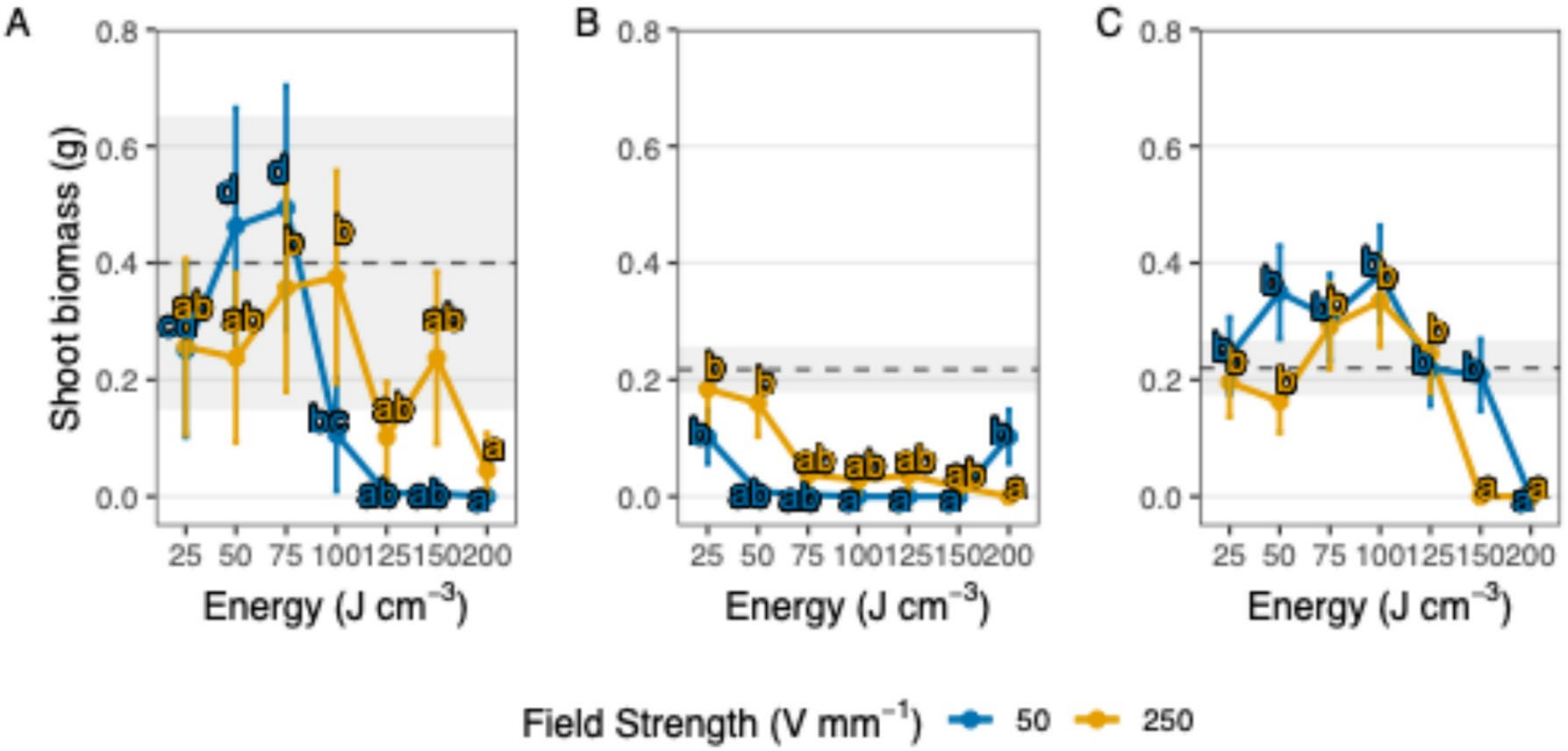
Shoot biomass of *Cyperus esculentus* (**A**), *Digitaria sanguinalis* (**B**), and *Echinochloa crus-galli* (**C**) following PEF treatment using the parallel plate applicator. Propagules were exposed to energy densities from 0 to 200 J cm^−3^ at field strengths of 50 and 250 V mm^−1^. Different letters indicate significant differences among treatments (*p* < 0.05). Model means ± SE (*n* = 8) are pooled from two studies. The dashed line and shaded area represent the mean and 95% confidence interval of the nontreated control.

Pulsed electric field treatment using the PP applicator significantly affected shoot biomass of *C. esculentus*, *D. sanguinalis*, and *E. crus-galli* (Fig. 6). For *C. esculentus* (Fig. 6A), energy density, field strength, and their interaction significantly affected biomass (*p* < 0.01). Biomass remained similar to the nontreated control at lower energy densities and declined sharply at ≥100 J cm^−3^, with greater suppression at 50 V mm^−1^ than at 250 V mm^−1^. Complete biomass elimination occurred at high energy densities when treatments were applied at 50 V mm^−1^, whereas biomass suppression remained incomplete at 250 V mm^−1^. For *D. sanguinalis* (Fig. 6B), biomass declined progressively with increasing energy density (*p* < 0.01), with reductions beginning at lower energy levels under 50 V mm^−1^ compared with 250 V mm^−1^.

In contrast, *E. crus-galli* (Fig. 6C) was less responsive to PEF, with biomass remaining similar to or greater than the control at lower energy densities and declining only at higher energy levels (≥150 J cm^−1^). These results indicate that weed species differed in sensitivity to PEF, with *C. esculentus* and *D. sanguinalis* responding at lower energy thresholds than *E. crus-galli*.

### Effect of PEF on plant-parasitic nematodes

**Fig. 7:**
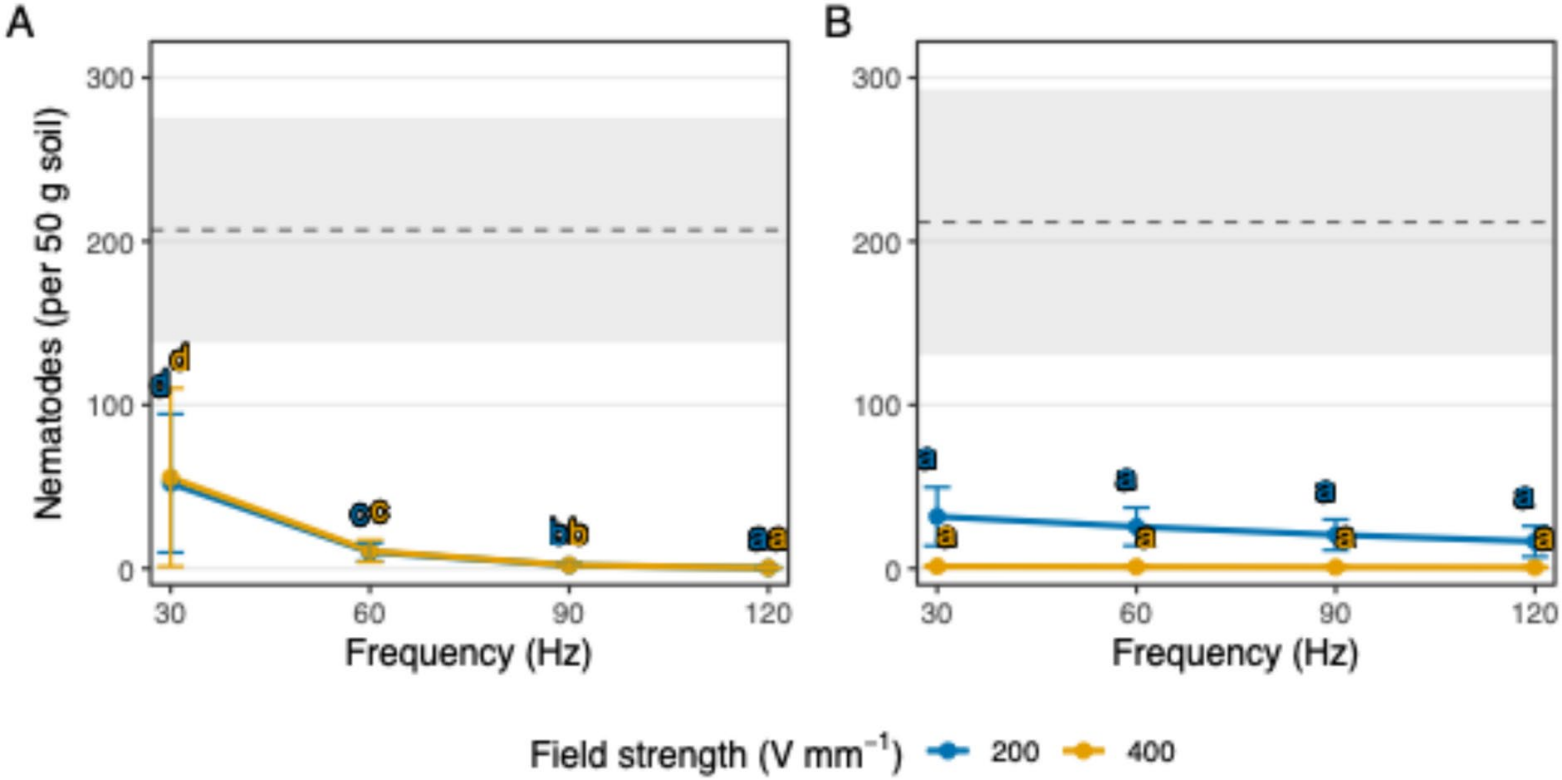
Response of *Meloidogyne chitwoodi* (**A**) and *Pratylenchus neglectus* (**B**) to PEF treatments at field strengths of 200 and 400 V mm^−1^ and frequencies of 0–120 Hz using the parallel plate applicator. Different letters indicate significant differences among treatments (*p* < 0.05). Model means ± SE (*n* = 8) are pooled from two studies. The dashed line and shaded area represent the mean and 95% confidence interval of the nontreated control.

Treatments with PEF significantly reduced plant-parasitic nematode densities compared to the nontreated control (Fig. 7). For *M. chitwoodi* (Fig. 7A), suppression increased with both frequency (*p* < 0.01) and field strength (*p* < 0.05), with densities declining from 30 to 60 Hz and reaching complete suppression at 90–120 Hz. A zero-inflated negative binomial model provided the best fit for these data (ΔAIC = 11.1). For *P. neglectus* (Fig. 7B), density was strongly influenced by field strength (*p* < 0.001), whereas frequency had no significant effect (*p* = 0.98). Increasing field strength from 200 to 400 V mm^−1^ substantially improved suppression, with reductions exceeding 98% at all frequencies. These results indicate that frequency strongly influenced *M. chitwoodi* suppression, whereas field strength exerted a greater influence on *P. neglectus control*.

### Effect of PEF on soilborne pathogens

**Fig. 8:**
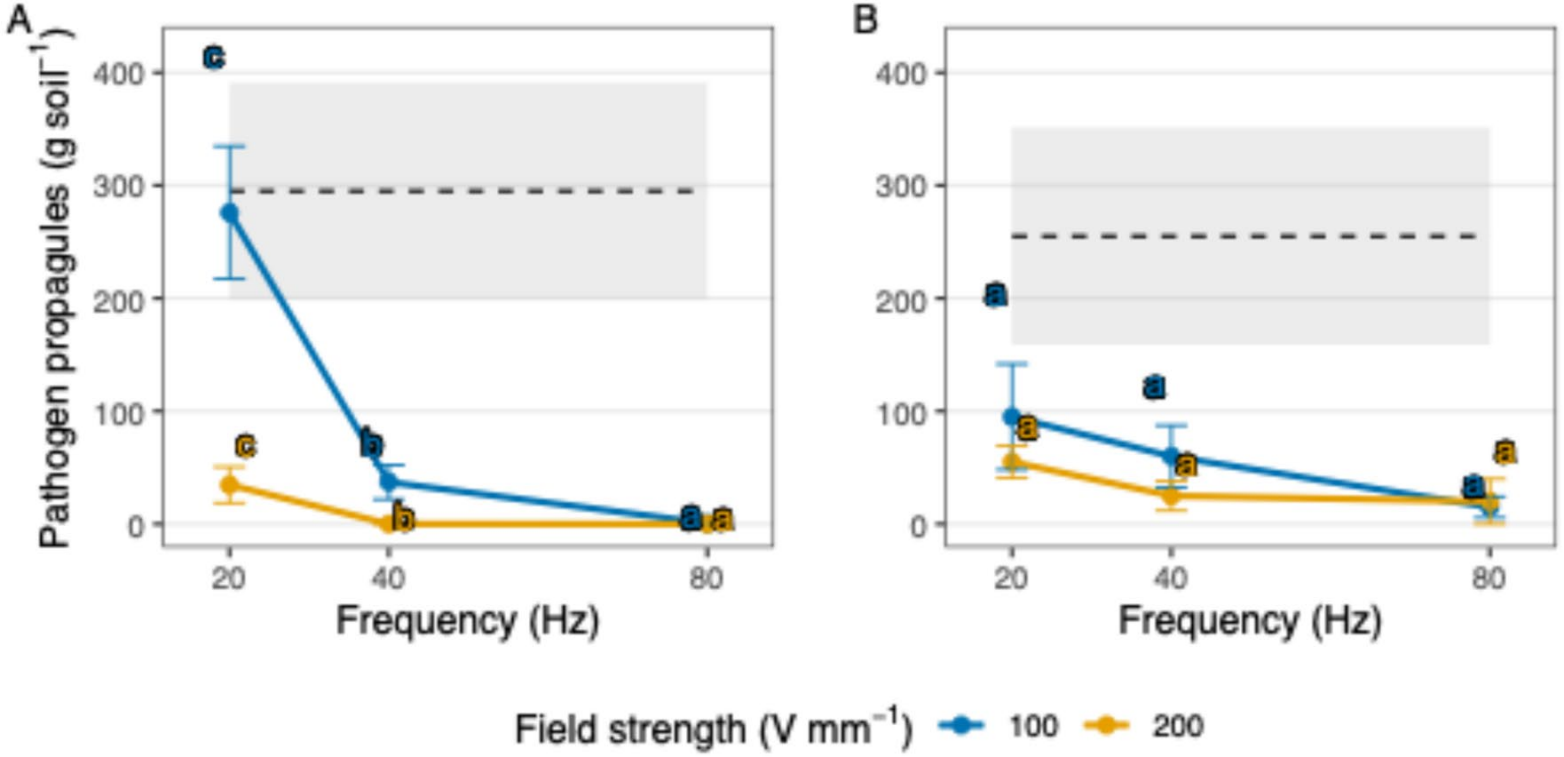
Response of *Phytophthora cinnamomi* (**A**) and *Phytophthora plurivora* (**B**) to PEF treatments at field strengths of 100 and 200 V mm^−1^ and frequencies of 0–80 Hz using the parallel plate applicator. Different letters indicate significant differences among treatments (*p* < 0.05). Model means ± SE (*n* = 8) are pooled from two studies. The dashed line and shaded area represent the mean and 95% confidence interval of the nontreated control.

**Fig. 9:**
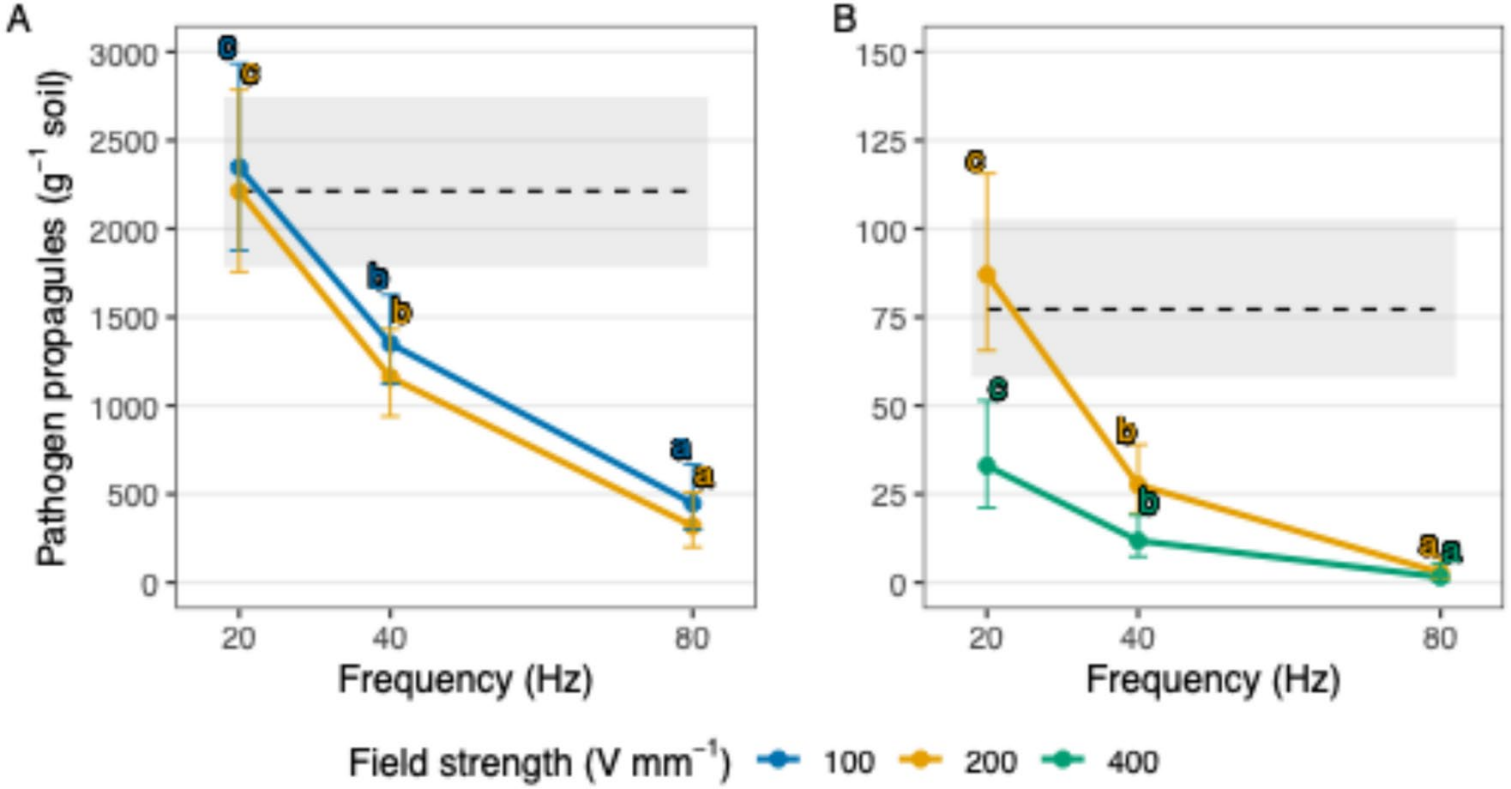
Response of *Verticillium dahliae* to PEF treatments applied at field strengths of 100 and 200 V mm^−1^ (**A**) and 200 and 400 V mm^−1^ (**B**) across frequencies (Hz) using the parallel plate applicator. Model means ± SE (*n* = 8) are pooled from two studies. The dashed line and shaded area represent the mean and 95% confidence interval of the nontreated control.

Field strength and frequency influenced PEF efficacy against all three soilborne pathogens (Figs. 8 and 9). For *P. cinnamomi* (Fig. 8A), suppression was driven by both frequency and its interaction with field strength (*p* < 0.05), with propagule counts reduced to nondetectable levels at 80 Hz under both 100 and 200 V mm^−1^. For *P. plurivora* (Fig. 8B), all treatments reduced propagule counts relative to the nontreated control. Field strength was the primary driver of suppression (*p* < 0.001), whereas frequency alone had a limited effect, although a significant interaction indicated that the effect of field strength depended on pulse frequency.

In contrast, *V. dahliae* (Fig. 9) responded primarily to frequency (p < 0.001), with little effect of field strength alone. The strongest suppression occurred at 80 Hz, particularly when combined with higher field strength. Differences in response at 200 V mm^−1^ between panels reflect differences in experimental conditions and organism-specific sensitivity rather than experimental inconsistency. These results indicate that pathogen responses to PEF are species-specific, with frequency playing a dominant role for *V. dahliae* and *P. cinnamomi*, and field strength being more important for *P. plurivora*.

## Discussion

Pulsed electric field-induced pest suppression is primarily attributed to electroporation^14,15^, which occurs when the applied electric field exceeds the critical transmembrane potential of biological membranes, resulting in reversible or irreversible membrane disruption and loss of cellular homeostasis. Electroporation can be reversible, in which cells recover from temporary membrane disruption, or irreversible, in which membrane damage leads to loss of viability. This outcome depends on field strength, pulse intensity, duration, number, and total energy input. Because this mechanism is not species-specific and affects a wide range of biological cells, PEF treatments have been explored as a non-chemical approach for inactivating microorganisms, plant tissues, and other biological structures^6,7,17^. In soil systems, this mechanism provides a basis for the simultaneous suppression of weeds, nematodes, and soilborne pathogens observed in this study. Building on earlier work by Riga et al.^5^, the present study shows that treatment efficacy is strongly influenced by applicator design and the resulting distribution of electrical energy within the treated soil.

Across experiments, applicator design, energy density, field strength, and pulse frequency jointly influenced treatment outcomes. Among these factors, applicator design emerged as the dominant determinant of efficacy. Increasing energy input resulted in effective suppression only when delivered through the parallel-plate (PP) applicator, whereas the vertical-probe (VP) applicator produced inconsistent responses even at higher energy densities. This outcome likely reflects differences in the electric field distribution within the soil volume. Numerical simulations and experimental studies have demonstrated that pulsed electric field chamber geometry strongly influences field uniformity and that the effective electric field strength within a treatment zone often deviates from theoretical estimates derived from electrode spacing. Treatment chambers may also contain transition zones where the electric field is insufficient to induce electroporation yet still contributes to overall energy input and heating^18–20^. Consequently, systems with uneven field distribution may deliver substantial energy without consistently reaching the threshold required for biological inactivation. The strong suppression observed with the PP applicator therefore likely reflects more uniform field delivery and greater soil volume exposed to effective electric field intensities.

Pulse frequency significantly enhanced nematode suppression, with higher frequencies resulting in greater mortality. Field strength also contributed to suppression, although its effect was more modest and consistent across treatments, particularly under the PP configuration. Previous studies have similarly identified electric field strength as a key parameter governing PEF treatment efficiency, with microbial and cellular inactivation occurring only when the applied field exceeds the electroporation threshold^11,21^. Interaction analyses indicated that increases in energy input and field strength were associated with improved biological control, particularly when combined with uniform electric field delivery, highlighting the integrated nature of PEF process parameters. Although cumulative energy density was held constant across several experiments, altering field strength changed the instantaneous electrical intensity experienced by biological tissues, which likely influenced whether electroporation thresholds were exceeded.

Weed responses to PEF were species dependent. *D. sanguinalis* was highly susceptible, exhibiting near-complete biomass suppression at intermediate to high energy densities regardless of field strength. *C. esculentus* showed moderate sensitivity; biomass reductions became pronounced at higher energy inputs, particularly under elevated field strength. In contrast, *E. crus-galli* was the species most tolerant to PEF; it maintained biomass at low to moderate energy densities and responded primarily to the highest doses. Previous studies have shown that PEF exposure can produce contrasting responses in plant tissues depending on treatment intensity and duration. At relatively low electric field strengths, PEF has been reported to stimulate seed germination and early seedling growth in several crop species, including lettuce, wheat, tomato, chickpea, and barley^22^. Previous studies suggest that these stimulatory effects may be associated with enhanced membrane permeability, increased enzymatic activity, and improved metabolic processes in germinating seeds. In contrast, higher electric field strengths or longer pulse durations often lead to germination inhibition or cellular damage due to irreversible electroporation and disruption of membrane integrity. For example, lettuce seedlings exhibited growth stimulation at 20–100 V mm^−1^ but inhibition at higher intensities, while *Arabidopsis thaliana* L. and *Triticum aestivum* L. showed similar intensity-dependent responses^22^. No previous reports were identified for PEF effects on *C. esculentus* tubers. Previous work on *C. esculentus* seeds exposed to repetitive PEF reported reduced germination at substantially higher field strengths (600–3750 V mm^−1^) than those applied in the present study (50–400 V mm^−1^)^23^. This difference suggests that effective suppression of underground propagules may depend not only on peak field strength but also on pulse duration, cumulative energy delivery, and soil electrical properties. These findings are consistent with electroporation theory, which predicts that biological responses vary with cell size and tissue characteristics, as well as the distribution of the applied electric field^24,25^. The variability in weed response observed in this study indicates that PEF is unlikely to provide a universal solution across all weed species. Instead, its effectiveness will depend on species-specific sensitivity, propagule type, growth stage, and application parameters. Therefore, PEF may be best implemented as part of an integrated weed management strategy, with treatment settings optimized for target species and combined with complementary practices where needed.

Overall, PEF suppressed plant-parasitic nematodes, soilborne pathogens, and weed propagules, although responses differed among organism groups and depended on applicator design and electrical parameters. Nematodes and pathogens were generally more sensitive and required lower energy inputs than weed propagules, suggesting that soil-applied PEF may be particularly effective for microbial suppression. In contrast, effective weed control may require direct exposure of aboveground tissues and optimized exposure strategies under field conditions.

For plant-parasitic nematodes and soilborne pathogens, field strength was among the most influential parameters affecting survival and viability, with the strongest suppression generally observed at 400 V mm^−1^. Similar relationships have been reported for microbial inactivation by PEF, where field strength governs the magnitude of induced membrane potentials and determines whether reversible or irreversible electroporation occurs^5,6,21^. Pulse frequency further enhanced suppression of *M. chitwoodi*, whereas responses of *P. neglectus* were less consistent, with partial survival observed at lower field strengths. The observed differences between *M. chitwoodi* and *P. neglectus* may also be influenced by the use of mixed developmental stages for *P. neglectus*, whereas only second-stage juveniles were evaluated for *M. chitwoodi*. Because stage-specific responses were not quantified, it is not possible to determine which stages were most susceptible to PEF. Therefore, the variability in response likely reflects both differences in stage composition and intrinsic biological characteristics.

Pulsed electric field treatments suppressed *Phytophthora* spp. and *V. dahliae*, with near-complete inactivation observed at higher field strength and frequency combinations (Figs. 8 and 9). This response is consistent with previous studies showing that PEF effectively inactivates soilborne fungal pathogens. For example, Chen et al.^26^ reported increased mortality of *Fusarium oxysporum* in greenhouse soils, reaching approximately 90–99% lethality when electric field strength increased from 5 to 14 kV cm^−126^. *V. dahliae* forms microsclerotia and *Phytophthora* species produce oospores and chlamydospores, which enable long-term survival in soil and contribute to their persistence under conventional management practices^27^; the strong response observed here suggests that PEF could be a promising non-chemical soil disinfestation strategy for both plant pathogenic genera.

Increasing applied energy elevated soil temperature and electrical conductivity, reflecting enhanced ionic mobility during treatment. Moderate temperature increases may contribute to membrane destabilization and biological damage, as mild heating is known to enhance the effectiveness of PEF treatments. Despite these physical changes, soil chemical properties remained largely unaffected, indicating that PEF application up to 200 J cm^−3^ did not substantially alter bulk nutrient availability. This likely reflects the short treatment duration used in this study, which limited physicochemical changes to detectable levels. PEF is known to alter soil physicochemical properties when treatment durations are prolonged (days), leading to ion electromigration, diffusion, and altered soil pH^1^. Effective suppression of plant-parasitic nematodes and soilborne pathogens was achieved at substantially lower energy inputs (25 J cm^−3^), suggesting that biological control may be achievable with short durations and relatively low energy requirements. At higher energy densities, localized heating and structural changes may occur, although such conditions are unlikely to be economically viable in commercial systems. From a sustainability perspective, PEF represents a non-chemical alternative that avoids chemical residues and may reduce environmental impacts associated with soil fumigation. However, its practical adoption will depend on energy requirements, equipment costs, and application efficiency relative to existing management practices. While these results demonstrate biological efficacy under controlled conditions, further research is needed to evaluate cost–benefit and feasibility at field scale. The timing of PEF application is likely to differ among target organisms. Pre-plant treatments may be most effective for reducing populations of soilborne pathogens and plant-parasitic nematodes, whereas weed control may require post-emergence or plant-targeted applications to ensure sufficient exposure of aboveground tissues. As a result, a single application timing may not optimize control across all pest groups. Integrated or sequential application strategies may therefore be necessary to maximize efficacy in field conditions^28,29^.

While this study demonstrates the potential of PEF for suppressing soilborne pests under controlled conditions, several factors require further investigation for field-scale application. The selectivity of PEF treatments with respect to crop species and non-target organisms, including beneficial soil microbes and invertebrates, remains an important consideration. Treatment efficacy is also likely influenced by environmental variables such as soil moisture, texture, and electrical conductivity, which can alter current distribution and energy transfer in soil. In addition, the effective depth of treatment and uniformity of energy delivery under field conditions will depend on applicator design and soil heterogeneity. Responses differed substantially among organism groups, with nematodes and soilborne pathogens generally requiring lower energy inputs than weed propagules, indicating that treatment strategies will likely require species-specific optimization of treatment parameters. Future research should therefore evaluate crop responses, non-target impacts, application timing, and field-scale performance to better define the practical implementation of PEF as a soil disinfestation strategy. Accordingly, the present study should be viewed as a proof-of-concept investigation demonstrating the biological potential of soil-applied PEF under controlled conditions, with additional validation needed before field-scale implementation.

## Methods

### Plant-parasitic nematode inoculum preparation

*Meloidogyne chitwoodi* inoculum, originally collected from a potato field in Hermiston, OR, was reared on tomato (*Solanum lycopersicum* ‘Rutgers’) in the greenhouse for 60 days. The tomato plants were harvested, and egg masses on roots were hand-picked into a scintillation vial containing water^30^. A 0.05% NaCl solution was then added to the vial and shaken for 3 min. The egg suspension was poured over nested 250- and 25-μm sieves, rinsed with water, and the eggs retained on the 25-mm sieve. The collected eggs were either used directly in experiments or placed in a hatching chamber^30^ to obtain second-stage juveniles (J2) after 5 to 7 days. *M. chitwoodi* J2 were collected daily and held at 4°C until used in experiments. *Pratylenchus neglectus* inoculum was originally collected from a wheat field in Pendleton, OR, and maintained on wheat (*Triticum aestivum* ‘Scarlett’) in a greenhouse. To obtain *P. neglectus* for experiments, plants were destructively harvested, roots washed free of soil, and then placed under intermittent mist (15-sec mist every 2 min) for five days^31^. The collected nematodes were held at 4°C until use. Suspensions containing plant-parasitic nematodes were adjusted to achieve desired inoculation densities.

### Soilborne pathogen inoculum preparation

*Phytophthora cinnamomi* (isolate R056) and *P. plurivora* (isolate R003) inocula were prepared using previously published methods^31^. Isolates were cultured on 20 mL of clarified V8 juice agar (composed of 3.4 g CaCO_3_ mixed with 340 mL of filtered V8 juice, diluted 1:4 with distilled water, and supplemented with 17 g of agar L^−1^) for seven days. The colonized agar was then cut into 1.5 cm^2^ pieces and added to a spawn bag (Fungi Perfecti, Olympia, WA) containing 2 L of clarified V8 juice broth (made from 150 mL of clarified V8 juice, CaCO_3_, and 1,850 mL of distilled water) mixed with 3 L of dry coarse vermiculite that had been autoclaved three times at 48-hour intervals. Spawn bags were incubated in the dark at 20 °C for six weeks, with weekly mixing for aeration. Inoculum density was assessed by dilution plating onto PARP (pimaricin, ampicillin, rifampicin, pentanitrochlorobenzene), a semi-selective medium for pythiaceous species^32^, and then stored at 20 °C until used within 1 week in experiments.

*Verticillium dahliae* inoculum was produced using a modified method from López-Escudero et al.^33^. A mixture of four isolates from raspberry were cultured on plates containing 20 mL of potato dextrose agar for six days at 24°C^6^. Conidia were washed from the surface with sterile distilled water, and 0.5 mL of the suspension was plated onto PDA covered with a sterilized disk of cellophane (Ultra Clear Cellophane, Research Products International, Mount Prospect, IL) and incubated for 14 days at 22°C. Microsclerotia were harvested from the plates and mixed with sterilized sand, then air-dried for 48 hours at 28°C. Inoculum density was estimated using an Andersen sampler^34^ to distribute 0.05 g of infested sand on ten plates of NP10^35^, a semi-selective medium for *V. dahliae*. Inoculum was stored at 20°C until used within 1 week in experiments.

#### Weed propagule inoculum preparation

Seeds of *D. sanguinalis*, *E. crus-galli*, and tubers of *C. esculentus* were collected from field populations at the OSU Lewis Brown Farm in Corvallis, OR, US. Propagules were manually cleaned to remove any foreign material and stored at room temperature in paper bags until used. Before use in experiments, seeds and tubers were placed in 5 °C water for 12 h.

#### Soil and experimental conditions

Sand and loam soils were purchased locally and combined (1:1, v/v) to create a uniform soil mixture. The soil was pasteurized prior to use using an autoclave (121 °C for 30 min) to reduce background biological activity. Soil samples were analyzed by the Oregon State University Soil Health Laboratory (Corvallis, OR, USA) using standardized protocols, including Mehlich-3 extraction for determination of macro- and micronutrients. The experimental unit consisted of a 10 cm by 10 cm plastic pot containing approximately 500 cm^3^ of soil. Treatments consisted of combinations of applicator type (VP and PP), field strength, and pulse frequency (Table 1). A nontreated control (inoculated soil without PEF application) was included for comparison. Experiments were arranged in a completely randomized design, with four replicates per treatment, and each experiment was repeated twice.

#### PEF delivery system

Pulsed electric field (PEF) treatments were delivered using a pulsed Direct Energy System (DES; Lisi Global Inc., Richland, WA, USA). The system generated high-fidelity pulsed DC square-wave outputs delivered between electrodes placed in soil. Pulse duration, frequency, and cumulative delivered energy were controlled for each treatment, and pulse-to-pulse variation was tightly constrained during each pulse train. We evaluated vertical-pin (VP) and parallel-plate (PP) electrode configurations (Fig. 1A, B). Each configuration produced distinct electric-field distributions while maintaining predetermined nominal field-strength targets (V mm^−1^). All treatments were applied to sterilized soil placed in square plastic containers with a top duration of 8.9 cm, a bottom width of 6.4 cm, and a depth of 8.9 cm. Containers were filled with approximately 500 cm^3^ (5 × 10^−4^ m^3^) of soil.

The vertical-pin applicator used in the small-pot experiments was a prototype designed for soil applications (Direct Energy System, Lisi Global Inc., Richland, WA, USA; Fig. 1A). The device consisted of two rows of four metallic pins with polarity assigned by row (one positive row and one negative row). Each pin was 0.00635 m in diameter and inserted into the soil during treatment. Within each row, pins were spaced at 0.0254 m, resulting in a treated swath of 0.0762 m. The treated soil volume was defined using the applicator-specific treatment footprint and insertion depth. For clarity, larger vertical-pin configurations used in field-scale work should be distinguished from the smaller vertical-pin applicator used in these pot trials where dimensions or results are discussed.

The PP applicator consisted of two aluminum plates (0.0508 × 0.0508 m; thickness 0.0031 m) placed in soil-filled pots (Fig. 1D–F). For biological treatments, plate spacing was set or measured for the individual treatment, and voltage was adjusted to achieve the prescribed nominal field strength (E = V/d), subject to the maximum DES output limit. The treated soil volume was defined as the treatment-specific inter-electrode soil volume between opposing plate faces. Considering only the inward-facing surface of each plate as the active electrode interface, the PP configuration provided a larger electrode-soil interface relative to the treated soil volume than the VP configuration.

We estimated the spatial distribution of electric potential and resulting electric field between electrodes using a numerical electrostatic model assuming a homogeneous and perfectly uniform medium. Under these conditions, the electric potential (ϕ) satisfies Laplace’s equation (∇^2^ϕ = 0), which describes the steady-state distribution of potential in the absence of internal charge sources. We calculated the electric field (E) from the potential gradient according to E = −∇ϕ, and the field magnitude was determined as |E| = √[(∂ϕ/∂x)^2^ + (∂ϕ/∂y)^2^]. The equations were solved on a two-dimensional grid using an iterative relaxation scheme based on weighted Jacobi/successive over-relaxation methods, with electrode regions treated as fixed-potential (Dirichlet) boundaries.

The model was used to compare relative geometry-driven field uniformity between applicator designs using fixed representative electrode geometries. It did not simulate transient pulse dynamics, soil heterogeneity, electrode contact resistance, moisture gradients, evolving soil conductivity, heating, or electrochemical effects. Therefore, simulated fields were used to interpret relative applicator behavior rather than to predict absolute delivered energy or local temperature rise.

For the PP simulation, electrodes were modeled as two fixed parallel boundaries separated by 5 cm. Boundary conditions were defined such that ϕ(0,y) = +V/2 and ϕ(W,y) = −V/2, where W represents the modeled electrode spacing. Under these idealized conditions, the analytical solution is linear, producing a uniform electric field between the plates given by |E| = V/W. Target electric field gradients were imposed by adjusting the applied voltage difference (ΔV) according to ΔV = (V mm^−1^) × 50 mm, with electrode potentials assigned as ±ΔV/2.

Pin electrodes were represented numerically as fixed-potential circular regions within the computational grids, corresponding to cylindrical electrodes arranged in two opposing columns. Solving Laplace’s equation numerically with these embedded boundary conditions provided the electric potential distribution. Because Laplace’s equation is linear, the normalized potential field was scaled to the desired applied voltage difference according to ϕ_scaled = ϕ_norm × (ΔV/2). Electric field components were estimated using central finite-difference approximations of the potential gradients, where Ex ≈ −(ϕᵢ₊₁,ⱼ − ϕᵢ₋₁,ⱼ)/(2Δx) and Ey ≈ −(ϕᵢ,ⱼ₊₁ − ϕᵢ,ⱼ₋₁)/(2Δy). The resulting electric field magnitude (V mm^−1^) was used to visualize spatial variation in field intensity across the treatment zone.

Energy density was defined as the total electrical energy delivered normalized to the treated soil volume (J cm^−3^). Delivered energy was calculated by integrating the measured voltage and current waveforms over each pulse according to E_pulse = ∫V(t) I(t) dt, where V(t) is voltage (V) and I(t) is current (A) during the pulse. Cumulative delivered energy was calculated by summing pulse energy across the pulse train and dividing by the treatment-specific soil volume. For each replicate, voltage and pulse-count settings were adjusted as needed to achieve the target nominal field strength and cumulative energy density for the corresponding electrode spacing, treated soil volume, and soil electrical response. Applied energy densities ranged from 25 J cm^−3^ for nematodes and soilborne pathogens to 200 J cm^−3^ for weed seeds and tubers (Table 1).

### Experimental procedures

The VP and PP applicators were evaluated for energy delivered to weed seeds and tubers at variable energy densities and field strengths (Table 1). Pulse frequency was held at 100 Hz, and pulse duration was held at 0.005 s unless otherwise indicated. Twenty seeds of *D. sanguinalis* and *E. crus-galli* were separately planted in soil-mix-filled pots at a depth of approximately 1 cm. Six tubers of *C. esculentus* were similarly planted at a depth of approximately 1 cm. The pots were moved to the greenhouse after treatment, and weeds were allowed to grow for eight weeks. The aboveground portion of the plant was harvested, and the roots rinsed free of soil under running tap water. All plant material was placed in a 70 ºC oven for 3 days, then weighed to determine the plant’s dry weight.

See Table 1 for our evaluation of the effect of the VP and PP applicators on plant-parasitic nematodes, with energy applied at the noted field strengths and frequencies. Total delivered energy density was held constant at 25 J cm^−3^ of soil, and pulse duration was held at 0.005 s unless otherwise indicated. Pots were filled with the soil mix, and 1,000 *M. chitwoodi* J2 or mixed stages of *P. neglectus* were added to three 2 cm deep holes in each pot in approximately 5 mL water. Energy was applied following the specific profile for each nematode (Table 1). After treatment, the soil in the pots was placed into resealable plastic bags, thoroughly mixed, and then moved to the laboratory for analysis. To evaluate the efficacy of PEF on *M. chitwoodi* and P*. neglectus*, 50 g of the soil sample was placed on a Baermann funnel for 5 days36. Plant-parasitic nematodes collected from funnels were counted using an inverted microscope at 20-40x magnification.

Only the PP applicator was used in experiments with soilborne pathogens; energy was delivered at the field strengths and frequencies listed in Table 1. Total delivered energy density was held constant at 25 J cm^−3^ of soil, and pulse duration was held at 0.005 s unless otherwise indicated. *Verticillium* and *Phytophthora* pathogen inocula were mixed separately into the dry soil mix to achieve a final concentration of ≥ 75 propagules per gram of soil (ppg). Infested soil was then distributed into pots for treatment. After treatment, efficacy was evaluated by dilution plating (for Phytophthora species) or using an Andersen sampler (for *V. dahliae*), as described above.

We also evaluated the effect of the PP applicator on soil physical and chemical properties. Soil, adjusted to a previously determined optimal moisture content for electrical conductivity, was placed in containers. Energy densities from 1 to 200 J cm^−3^ were applied, and temperature (°C) and electrical conductivity (S m^−1^) were recorded immediately after each application. A K-type thermocouple probe was used to simultaneously assess heat generation and ionic mobility. For chemical characterization, soil samples were analyzed using the Mehlich-3 extraction method, which enables the simultaneous determination of macro- and micronutrients, by a certified commercial laboratory following standardized protocols for sample preparation, extraction, and quantification. The resulting dataset included concentrations of Ca, Mg, K, P, Fe, Mn, Zn, and Cu, expressed in mg kg^−1^ of soil.

### Data analysis

All analyses were performed using R software (version 4.5.3; R Core Team, Vienna, Austria). Data were analyzed using linear mixed-effects models for each response variable. Field strength and frequency were included as fixed effects, and their interaction was tested, with block (and study, when applicable) included as random effects. Model assumptions of normality and homoscedasticity were evaluated using residual diagnostics (including visual inspection of residual and Q–Q plots), and data were transformed when necessary to meet these assumptions. Post-hoc comparisons were conducted using Tukey’s honest significant difference (HSD) test (*p* < 0.05).

Soil chemical data (Mehlich-3) were analyzed by principal component analysis (PCA) using the prcomp() function after centering and scaling. Differences among energy densities (0, 50, and 200 J cm^−3^) were assessed by ANOVA with Tukey’s test (*p* < 0.05). A permutational multivariate analysis of variance (PERMANOVA, 999 permutations, Euclidean distance) and redundancy analysis (RDA) were also performed.

## Supplementary Information

Supplementary Information.

## Data Availability

The data that support the findings of this study are available from the corresponding author upon reasonable request.
