# Supplementary material for: Field strength and frequency-dependent effects of pulsed electric fields on soilborne nematodes, pathogens, and weeds
Source: Sci Rep. 2026 Jun 8;16:25964. doi: 10.1038/s41598-026-56820-y (PMC13487166; doi:10.1038/s41598-026-56820-y)
Supplement: Supplementary file 1 [file 41598_2026_56820_MOESM1_ESM.docx]

| Supplementary  **Table 1. Summary of analysis of variance for significant factors and interactions** | | | | | | |
| --- | --- | --- | --- | --- | --- | --- |
|  | *Cyperus esculentus* | | *Digitaria sanguinalis* | | *Echinochloa crus-galli* | |
| *Factors* | χ² | *P-value* | χ² | *P-value* | χ² | *P-value* |
| *E* | *99.62* | ***** | 7.36 | *** | 16.94 | *** |
| *FS* | *8.83* | **** | 7.31 | ** | 8.04 | ** |
| *E * FS.* | *26.15* | ***** | 3.83 | ** | 3.18 | * |
| *Phytophthora* | | | | | | |
|  | *P. cinnamomi* | | *P. plurivora* | |  |  |
| *FS* | *5.99* | *0.014* | *15.67* | *<0.001* |  |  |
| *FQ* | *9.06* | *0.002* | *0.82* | *0.36* |  |  |
| *FS * FQ* | *30.08* | *<0.001* | *6.14* | *0.013* |  |  |
| *Pathogen* | | | | | | |
|  | *Verticillium S1* | | *Verticillium S2* | |  |  |
| *FS* | *4.05* | *0.044* | *0.06* | *0.79* |  |  |
| *FQ* | *86.28* | *<0.001* | *46.97* | *<0.001* |  |  |
| *FS * FQ* | *7.39* | *0.006* | *7.52* | *0.006* |  |  |
| *P – probability: 0 ‘***’ 0.001 ‘**’ 0.01 ‘*’ 0.05 ‘.’ 0.1 ‘ ’ 1*  *Abbreviations –* FS – nominal field strength target; FQ – pulse frequency; PD – pulse duration; E – Energy density | | | | | | |
